# Murine Methyl Donor Deficiency Impairs Early Growth in Association with Dysmorphic Small Intestinal Crypts and Reduced Gut Microbial Community Diversity

**DOI:** 10.1093/cdn/nzy070

**Published:** 2018-10-03

**Authors:** Antonio V Alves da Silva, Stephanie B de Castro Oliveira, Sara C Di Rienzi, Kathleen Brown-Steinke, Lauren M Dehan, Jill K Rood, Vinicius S Carreira, Hung Le, Elizabeth A Maier, Kristina J Betz, Eitaro Aihara, Ruth E Ley, Geoffrey A Preidis, Lanlan Shen, Sean R Moore

**Affiliations:** 1 Instituto de Biomedicina, Universidade Federal do Ceará, Fortaleza, Brazil; 2 Division of Gastroenterology, Hepatology, and Nutrition, Department of Pediatrics, Cincinnati Children's Hospital Medical Center, Cincinnati, OH; 3 Department of Molecular Virology and Microbiology, Baylor College of Medicine, Houston, TX; 4 Vet Path Services, Mason, OH, USA; 5 Department of Pharmacology and Systems Physiology, University of Cincinnati College of Medicine, Cincinnati, OH, USA; 6 Department of Microbiome Science, Max Planck Institute for Developmental Biology, Tübingen, Germany; 7 Division of Gastroenterology, Hepatology, and Nutrition, Department of Pediatrics, Baylor College of Medicine Houston, TX; 8 USDA Children's Nutrition Research Center, Department of Pediatrics, Baylor College of Medicine, Houston, TX; 9 Division of Gastroenterology, Hepatology, and Nutrition, Department of Pediatrics, University of Virginia School of Medicine, Charlottesville, VA

**Keywords:** folic acid, choline, malnutrition, enteropathy, intestinal stem cell, DNA methylation, microbiome, enteroids

## Abstract

**Background:**

Folate and choline are essential methyl donor nutrients throughout the life span; however, the adverse effects of combined deficiency on early growth, intestinal epithelial morphology, and the gut microbiome remain only partially understood.

**Objectives:**

We investigated the effects of dietary folate and choline deficiency on early growth, small intestinal (SI) epithelial architecture, and the gut microbiota of mice. To explore potential mechanisms for adverse effects on gut epithelial morphology, we also evaluated gene expression and DNA methylation in mouse intestinal epithelial organoids (enteroids) maintained in methyl donor–deficient (MDD) conditions.

**Methods:**

Pregnant dams were administered 1 of 4 diets: *1*) control diet (CD−), *2*) an isocaloric MDD− diet, or *3*) CD+ and *4*) MDD+ formulations containing 1% succinylsulfathiazole to inhibit folate-producing gut bacteria. We weaned pups to their dams’ diet at 3 wk of age and monitored body weight and tail length pre- and postweaning. We measured serum folate, SI crypt morphology, and microbiota composition at 7 wk of age.

**Results:**

Both MDD+ and MDD− diets impaired early ponderal and linear growth, lowered serum folate concentrations, and produced patchy areas of increased crypt depth throughout the SI. Succinylsulfathiazole increased crypt depth independently of diet. MDD or succinylsulfathiazole, alone or in combination, altered the gut microbiome, with decreased Bacteroidales and Clostridiales, increased Lactobacillales and Erysipelotrichaceae taxa, and decreased α-diversity indexes. Enteroids maintained in MDD media displayed dysmorphic crypt domains, altered expression of stem cell and secretory differentiation genes, and decreased DNA methylation of the glycosylation genes Beta-1,4-N-Acetyl-Galactosaminyltransferase-1 (*B4galnt1*) and Phosphoethanolamine/Phosphocholine-Phosphatase (*Phospho1*).

**Conclusion:**

MDD impairs ponderal and linear growth in mice in association with dysmorphic SI crypts and reduced gut microbial diversity. In vitro methyl donor deficiency similarly induced dysmorphic crypts in mouse enteroids in conjunction with altered gene expression and DNA methylation.

## Introduction

Despite declining rates of folate deficiency worldwide, folate insufficiency remains a significant public health issue (1–3). Similarly, choline is abundant in common dietary constituents; however, ∼90% of individuals do not meet their adequate daily intake (4). Both folate and choline provide one-carbon units for the synthesis of the methyl donor *S*-adenosylmethionine, a ubiquitous component required for cell biosynthesis of a wide range of components that also plays a key role in DNA methylation, a fundamental epigenetic mechanism (5, 6). Further, both micronutrients are produced and consumed by gut microbiota; hence, animals not only obtain folate and choline from dietary sources but also share these nutrients with, and derive them from, resident gut microbes. Interest in adverse gut effects of deficiencies in folate and other methyl donor nutrients such as choline has risen in conjunction with greater appreciation of their interplay with epigenetics and the microbiome (7–9).

We have previously shown that weanling protein-energy malnutrition induces intestinal epithelial abnormalities that phenocopy some features of environmental enteropathy (EE) (10). More recently, Brown et al. (11) recapitulated additional features of EE in mice by modulating both diet and oral microbial exposures. Because folate is used as an adjunct therapy for tropical sprue (persistent diarrhea on a background of EE) and is also important for DNA methylation, we hypothesized methyl donor deficiency (MDD) would induce the growth failure and crypt hypertrophy seen in EE by modulating intestinal stem cell dynamics (12–14).

In the current study, we designed mouse experiments to investigate the effects of combined dietary folate and choline deficiency, without macronutrient restriction, on prenatal and postnatal growth, intestinal epithelial structure and function, and the gut microbiome. In addition, we tested the effects of choline and folate deficiency on crypt morphology, gene expression, and global DNA methylation in mouse small intestinal (SI) organoids. Here we report that: *1*) MDD impairs ponderal and linear growth in conjunction with patchy findings of dysmorphic SI crypts and reduced gut microbial diversity and *2*) in vitro MDD increases crypt length, alters the expression of genes involved in intestinal stem cell differentiation, and reduces DNA methylation of genes related to mucin production.

## Methods

### Murine model of MDD

Wild-type C57BL/6 timed pregnant mice with pups at 11–14 days of gestation were purchased from Jackson Laboratories and housed in a barrier facility with an ambient temperature of 22°C, a relative humidity ranging from 30% to 70%, and a 14:10-h light-dark cycle. Pregnant females were placed in separate cages with free access to water and randomly assigned to 1 of 4 experimental diets: *1*) control diet (CD−); *2*) control diet with succinylsulfathiazole 1% (10 g/kg) (CD+); *3*) methyl donor–deficient diet without succinylsulfathiazole (MDD−); and *4*) methyl donor–deficient diet with succinylsulfathiazole 1% (MDD+). These diets were continued through birth. Diets were associated with no clear group differences in litter size. Shortly after birth, litter sizes were adjusted to 5 or 6 pups/dam. Succinylsulfathiazole, an antibacterial sulfonamide that competes with the folate precursor para-aminobenzoic acid for the active site of the enzyme dihydropteroate synthase (15), was added to the diet to eliminate or decrease the amount of folate and choline produced by intestinal microbiota. Each diet was formulated to meet all nutrient requirements (except for folate and choline) and to be isocaloric and isonitrogenous (**Supplemental Table 1**) on the basis of the analysis provided by the manufacturer (Research Diets, Inc.). All diets were irradiated before use and consumed ad libitum. Before weaning, dams and pups were weighed 3 times/wk. On day-of-life 23, pups were weaned to their dams’ diet, and then weighed 2 times/wk. In addition, food consumption was measured weekly after weaning. Weanlings were caged together (≤4/cage), according to their experimental group and sex. Mice were allowed access to food and water until the morning of death. Male mice were killed at 7 wk of age by carbon dioxide inhalation and cervical dislocation. Blood samples were obtained by retro-orbital access and cardiac puncture for complete blood count and folate concentrations. Intestinal tissue along multiple segments of the SI was obtained for histological analysis. All mouse protocols were approved by, and performed in accordance with, the Institutional Animal Care and Use Committee of the Cincinnati Children's Hospital Medical Center.

### Intestinal morphometry

Cross-sections of duodenum, jejunum, and ileum were obtained at 2 cm and 8 cm from the gastric outlet and at 3 cm proximal to the cecum, respectively, to standardize the morphometric analysis. Segments were fixed in 4% formalin, embedded in paraffin, and stained with routine hematoxylin-eosin. Crypt dimensions and crypt density were measured in a blinded manner using Image J version 1.47 h (http://imagej.nih.gov/ij/) software. Nuclear morphology was assessed by measuring the length of the major axis of the nuclei, using NIS Elements version 4.5 software.

### Immunohistochemistry qPCR

For bromodeoxyuridine (BrdU) staining, mice were injected intraperitoneally with 1.0 mL of concentrated reagent/100 g body weight. SI tissue was fixed and embedded in paraffin, as described above. Sections were stained as per the manufacturer-suggested protocol (BrdU kit; Invitrogen). Images were digitally captured using a 3007A Nikon 90i upright microscope, and quantification of BrdU staining was performed in a blinded manner by counting the number of BrdU-positive cells per 20 crypts in 3 mice/group, using NIS Elements version 4.5 software.

### Folate assay and complete blood count

Mice were killed by carbon dioxide inhalation and cervical dislocation. Blood was collected by intracardiac puncture and centrifuged (2000 × *g* for 10 min at 4°C) to separate serum from RBCs. Serum samples were stored at −80°C for subsequent assays. Folate concentrations were measured by a specific ELISA (Cat# CEA610Ge, Cloud-Clone Corp.) according to the manufacturer’s instructions. Blood was collected from the retro-orbital plexus and immediately tested using an automated hematology analyzer (Hemavet 850; Drew Scientific) to obtain measurements of hemoglobin and mean corpuscular volume.

### Microbiome studies

#### Sample collection

Stools were collected from the cages of male mice at 6 wk of age (1–3 mice/cage). Harvest of jejunal tissue was performed at 7 wk of age. Stool and jejunal DNA were isolated using a QIAamp DNA extraction kit (QIAGEN). Each sample was immediately sealed in a cryo-tube and stored at −80°C until analysis.

#### 16S rRNA gene analysis

We processed, filtered, and analyzed the 16S rRNA amplicon data using QIIME (Quantitative Insights Into Microbial Ecology) version 1.9.0 (16). Paired-end reads were joined using join_paired_ends.py running the fastq-join method and requiring ≥200 bp of sequence overlap. Joined reads were demultiplexed using split_libraries_fastq.py requiring a Phred quality cutoff of 25 to remove ambiguous barcodes and low-quality reads. Reads were clustered into operational taxonomic units (OTUs) using open-reference OTU picking at 97% sequence identity to the Greengenes database version 13.8 (17).

For all subsequent analyses, we rarified data to 10,000 sequences/sample. α-Diversity was analyzed in QIIME using the alpha_diversity.py script to apply Faith's Phylogenetic Diversity, Shannon diversity index, Chao1 estimate of species richness, and the observed OTUs metric. Significant differences between groups were determined by pairwise 2-sided Wilcoxon tests and a false discovery rate (FDR) multiple testing correction with *P* < 0.05 considered as significant. β-Diversity was assessed using the UniFrac metric implemented in QIIME 1.9.0. We performed adonis [permutational multivariate analysis of variance (PERMANOVA), controlling for cage effects] with 10,000 iterations in QIIME using the compare_categories.py script. β-Diversity plots were made using the phyloseq package (18) with the ordplot function using a *t* distribution.

We identified OTUs differentiating samples by first filtering OTU tables to only include those OTUs present in ≥20% of the samples and with ≥1 sample having an average of 10 sequence counts across all samples. To the filtered OTU tables, we applied a Kruskal–Wallis test with a FDR cutoff of 10% using the group_significance.py script in QIIME. Heat maps on these OTUs passing at least an FDR < 0.1 were created using the make_otu_heatmap.py script in QIIME.

#### Effects of MDD on identity and methylation of intestinal stem cells in murine intestinal organoids

Intestinal organoids were prepared by isolating fresh mid-jejunal crypts using a previously described method (19). Briefly, the first proximal 4 cm of jejunum was isolated, washed, and treated with 2 mM cold EDTA for 30 min at 4°C and cut into 1-cm sections. The suspension was transferred into sucrose–sorbitol buffer, gently shaken for 2 min to release the intestinal crypts, and strained through 70-μm filters. After washing and centrifugation at 150 × *g* for 5 min at 4°C, the crypts were plated in a Corning Matrigel Growth Factor and reduced and incubated at 37°C to allow the polymerization of the Matrigel. Culture medium containing DMEM/F12, L-glutamine (200 nM), penicillin/streptomycin 1%, HEPES 10 mM, N2 1x, B27 1x, and the recombinant growth factors R-spondin (500 ng/mL), mouse Noggin (10 ng/mL), and mouse EGF (50 ng/mL) were added to the Matrigel. Enteroids were passaged weekly.

To study the effects of folate and choline deficiency, 2 types of culture media were used to maintain enteroids through 3 passages: medium containing regular concentrations of folate (2.65 mg/L) and choline (8.98 mg/L) found in regular DMEM/F12, or culture medium containing only 10% of these concentrations (folate: 0.27 mg/dL; choline: 0.9 mg/dL) prepared with a customized DMEM/F12.

#### Real-time PCR analysis

RNA was isolated from mouse intestinal organoids using an Aurum Total RNA Mini Kit (Bio-Rad) according to the manufacturer's instructions. cDNA was synthesized with the use of an iScrip Advanced cDNA Synthesis Kit (Bio-Rad). PCR commercial primers were acquired from Bio-Rad and are described in detail in **Supplemental Table 2**. PCR reactions were performed using the CFX384 real-time PCR detection system (Bio-Rad). The reference gene β-actin was used to normalize all qPCR gene expression, which was plotted relative to this control gene. Enteroid PCR data were generated from tissue obtained from 3 mice (2 replicates each) whose intestinal organoids were cultivated for 3 consecutive passages.

#### Whole genome and quantitative DNA methylation by bisulfite-sequencing

Whole genome DNA methylation profiling by bisulfite-sequencing was performed and analyzed according to a previously published protocol (20). Briefly, genomic DNA was isolated from the pool of 5 wells (∼480 enteroids) per group, following the instructions of the manufacturer of the DNA isolation kit (Qiagen). DNA was sonicated and adaptor-ligated genomic DNA was treated with sodium bisulfite by the EZ DNA Methylation-Direct kit (Zymo Research). The treated DNA was amplified (18 cycles) using adaptor-specific primers and fragments of 200–500 bp were isolated. The amount and size distribution of libraries were determined using the Pico Green fluorescence and the Agilent 2100 Bioanalyzer, respectively. Each library was sequenced as 100-bp paired-end reads. Base calling was performed using the standard Illumina pipeline. After removing adaptor sequences and low-quality tails, reads were mapped to the mouse reference genome.

Methylation at specific CpG gene regions was quantified by bisulfite-pyrosequencing using the PyroMark Q96 MD instrument (21). Sequencing assays and primers are summarized in **Supplemental Table 3**. Positive controls (CpG Mehylase M. SssI-treated genomic DNA) and negative controls (whole genome amplified genomic DNA) were included for each assay, mixing experiments to reduce bias, and repeated experiments to assess reproducibility. Annealing temperatures were optimized to overcome PCR bias as previously reported (22).

#### Statistical analysis

Statistical analyses were performed using GraphPad Prism version 7.0 (GraphPad) with bars indicating the mean ± SE. Comparisons of linear growth, weight, food consumption, food efficiency, crypt length, serum folate, hemoglobin, cell proliferation, and nuclear dimensions were made using 1-factor ANOVA with Bonferroni's posttest. Comparisons of gene expression and DNA methylation levels were made using *t* tests. For all analyses, a *P* value < 0.05 was considered to be statistically significant.

## Results

### Prenatal and postnatal MDD impairs growth in mice

**FIGURE 1:**
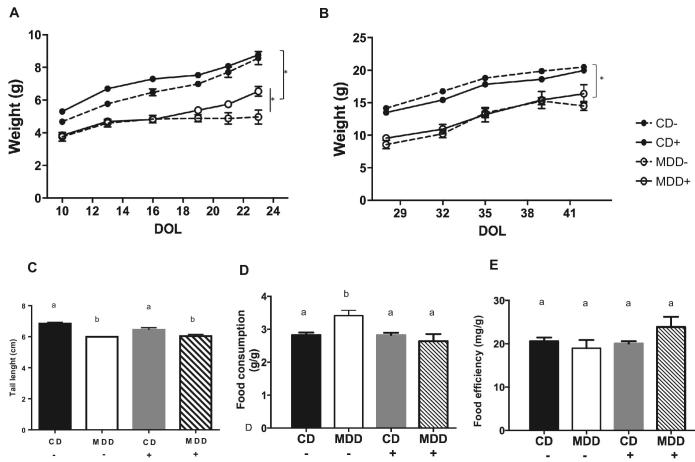
Prenatal and postnatal exposure to methyl donor deficiency diet leads to growth faltering in suckling and weaned mice. (A) Pups of dams fed MDD diets with or without antibiotics (*n* = 7 and *n* = 5, respectively) exhibited significant failure to thrive compared with pups of dams fed CD with or without antibiotics (*n* = 9 and *n* = 11, respectively). (B) Growth faltering persisted, without catch-up growth, until 7 wk of age. (C) Prenatal exposure to methyl donor deficiency with antibiotics impaired linear growth as measured by tail length. Weight differences were not explained by variation in (D) food consumption or (E) feed efficiency. Values are means ± SEMs. Means without a common letter differ, *P* < 0.05. CD+/−, control diet with/without succinylsulfathiazole; DOL, day of life; MDD+/−, methyl donor–deficient diet with/without succinylsulfathiazole. Asterisk indicates (*) *P* < 0.05 for group differences by repeated measures ANOVA with Bonferroni correction for multiple comparisons.

Administration of MDD diets (with or without succinylsulfathiazole) to pregnant dams was not associated with fetal loss; however, MDD pups were markedly underweight relative to CD pups, with the lowest weight value seen in MDD− (no succinylsulfathiazole) weanlings on day of life 23 (MDD−, **Figure 1A**). MDD+ pups gained weight more quickly than MDD− pups during suckling (Figure 1A, *P *< 0.05); however, these relative differences did not persist beyond weaning (Figure 1B). Upon weaning to their dams’ diet, both MDD+ and MDD− mice remained significantly underweight relative to CD+ and CD− mice, respectively (Figure 1B). In the CD groups, succinylsulfathiazole administration produced no detectable effects on weight gain during either of the suckling or postweaning periods. Tail length, a surrogate for linear growth, was significantly reduced in both MDD− and MDD+ groups compared with the CD− group (Figure 1C). These differences in postweaning weight and length were not accounted for by differences in either food consumption (Figure 1D) or feed efficiency (Figure 1E); however, MDD− mice displayed significant hyperphagia relative to the other groups (Figure 1D). These results indicate that MDD impairs fetal, postnatal, and weanling growth in mice.

### MDD diets induce folate deficiency and systemic manifestations thereof

**FIGURE 2:**
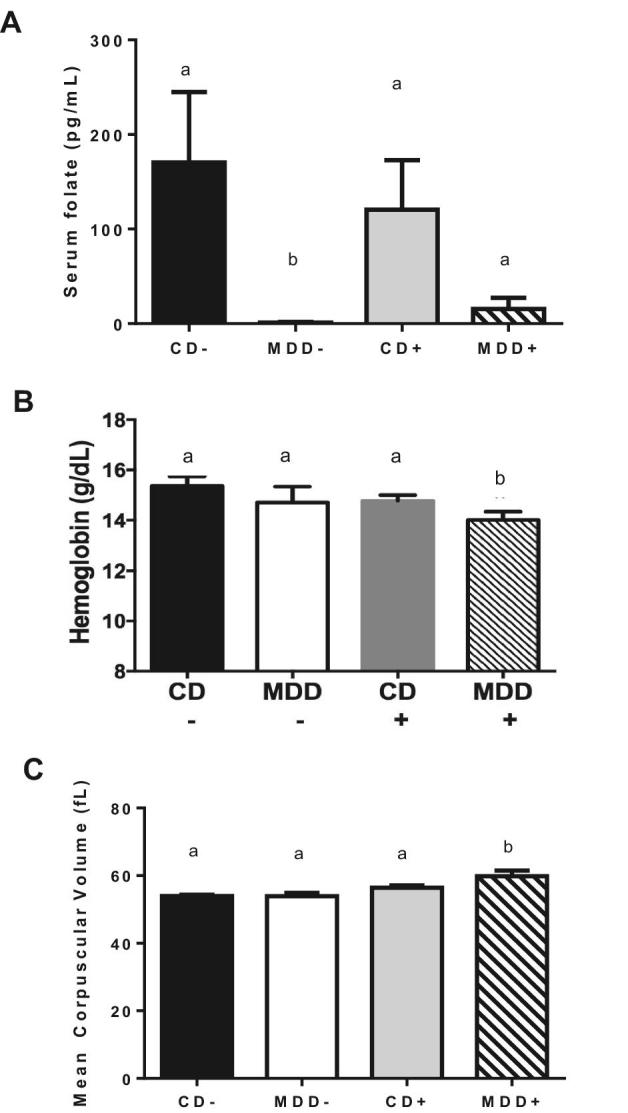
Combined prenatal and postnatal methyl donor deficiency leads to low serum folate and macrocytic anemia. (A) Prenatal and subsequent exposure to isocaloric diets lacking folate and choline induces folate deficiency, here represented by a decrease of >80% in the serum folate concentrations in both MDD groups compared with controls. Systemic manifestations of folate deficiency in the MDD+ group included (B) decreased hemoglobin concentrations and (C) increased mean corpuscular volume of RBCs. Values are means ± SEMs, *n* = 4–10. Means without a common letter differ, *P* < 0.05. CD+/−, control diet with/without succinylsulfathiazole; MDD+/−, methyl donor–deficient diet with/without succinylsulfathiazole.

We measured serum folate status by ELISA upon killing at 7 wk of age (**Figure 2A**). Both MDD+ and MDD− mice were folate deficient relative to CD mice. However, MDD+ mice alone exhibited systemic manifestations of folate deficiency as evidenced by a modest but statistically significant decrease in hemoglobin concentrations (Figure 2B) compared with CD− mice (*P* < 0.05) and an increase in the mean corpuscular volume of erythrocytes also compared with CD− mice (Figure 2C), suggesting the presence of megaloblastic anemia.

### Patchy crypt hypertrophy, intestinal epithelial cell proliferation, and elongation of nuclei

**FIGURE 3:**
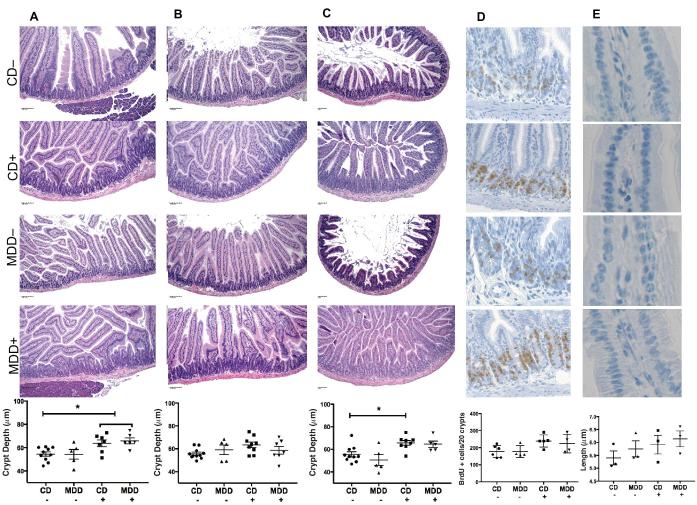
Methyl donor deficiency effects on crypt morphology, cell proliferation, and nuclear dimensions. Sections of duodenum (A), jejunum (B), and ileum (C) showing patchy areas of increased crypt depth. (D) Increased intestinal epithelial proliferation in both CD+ and MDD+ groups as evidenced by BrdU staining of jejunal sections. (E) Sections of jejunal midvillus regions were analyzed for nuclear morphology, with a trend towards nuclear lengthening in the CD+ and MDD+ groups. Values are means ± SEMs. *Significant difference (*P* < 0.05) between paired groups. BrdU, bromodeoxyuridine; CD+/−, control diet with/without succinylsulfathiazole; MDD+/−, methyl donor deficient diet with/without succinylsulfathiazole.

To determine the extent to which the body growth and systemic effects of MDD and antibiotic exposure were accompanied by enteropathy, we examined crypt/villus architecture, epithelial proliferation, and intestinal epithelial nuclear morphology along the length of the SI. In MDD+ and MDD− mice, we detected patchy areas of crypt elongation in all segments of the SI (**Figure 3**). To our surprise, in both the duodenum and ileum, CD+ mice exhibited significant crypt hypertrophy (Figure 3). The most consistent and remarkable crypt hypertrophy was seen in the ileum (**Supplemental Figure 1**), with the CD+ group showing a statistically significant difference compared with the CD− group (*P *< 0.05 by ANOVA). In the ileum of the CD+ and MDD+ groups, the morphologic changes were more remarkable in comparison with the CD− group: crypt elongation, an increase in the population of goblet cells, and a decrease in the population of Paneth cells. BrdU staining of jejunal tissue suggested an apparent increase in proliferative activity in both CD+ and MDD+ groups; however, this difference was not statistically significant. In a limited number of mice, we compared midvillus nuclear morphology between groups to look for megaloblastic changes. We observed a nonstatistically significant trend towards increasing nuclear length from the CD− group, followed by the MDD−, CD+, and ultimately the MDD+ group (Figure 3E). Taken together, these results indicate that MDD induced a patchy crypt hypertrophy but that succinylsulfathiazole had a more pronounced effect than MDD on both SI crypt length and intestinal epithelial proliferation.

### MDD and succinylsulfathiazole decrease intestinal microbiota community diversity

**FIGURE 4:**
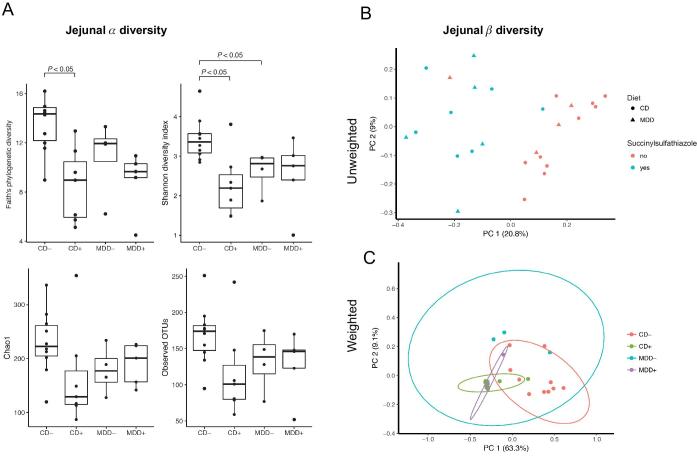
MDD diets or succinylsulfathiazole decrease jejunal microbiome diversity. (A) α-Diversity metric calculated by Faith's phylogenetic diversity, Shannon diversity index, Chao1, and observed OTUs. Significant differences between paired groups are indicated. (B, C) PC analysis on the unweighted (B) and weighted (C) Unifrac distance metrics. CD+/−, control diet with/without succinylsulfathiazole; MDD+/−, methyl donor–deficient diet with/without succinylsulfathiazole; OTU, operational taxonomic unit; PC, principal coordinate.

**FIGURE 5:**
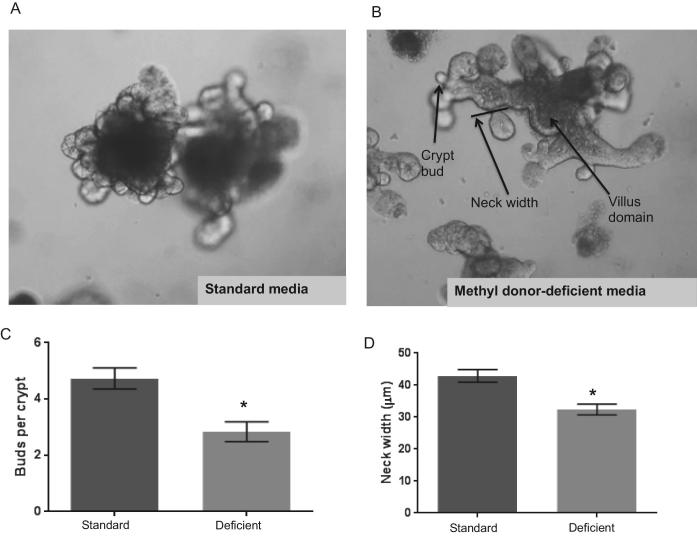
Methyl donor–deficient enteroids display deranged crypt morphology. Representative images of jejunal enteroids grown in either (A) standard media or (B) methyl donor–deficient media (containing 10% of standard concentrations of folate and choline) show dysmorphic features. Dysmorphologic features of methyl donor–deficient enteroids included a qualitative increase in crypt:villus length ratio, suggesting relative crypt hypertrophy; (C) a decreased number of buds per crypt domain in methyl donor–deficient enteroids compared with standard media; and (D) decreased neck width in crypt domains. Values are means ± SEMs (*n* = 6). *Significant differences between the groups at *P* < 0.05.

To determine whether the observed changes in body growth and SI crypt morphology in MDD and antibiotic-exposed mice corresponded to alterations in gut microbial communities, we measured the bacterial composition of jejunal homogenates and fecal pellets using 16S rRNA gene sequencing. In the jejunum, succinylsulfathiazole administration significantly decreased sample α-diversity in CD mice (CD+ compared with CD−, *P *< 0.05) but not MDD mice (MDD+ compared with MDD− mice), suggesting that there are few bacteria present on the methyl-deficient diet targeted by succinylsulfathiazole (**Figure 4A**). We detected no difference in α-diversity between the CD+ and MDD+ mice (Figure 4A). In contrast, α-diversity was greater in CD− than in MDD− mice by the Shannon diversity index, i.e., MDD shifted the gut microbiota in the absence of antibiotics (*P *< 0.05) (**Figure 5A**). To determine the compositional differences among the mouse groups, we assessed β-diversity using the unweighted UniFrac metric. We observed that samples separated by antibiotic treatment (Figure 4B, PERMANOVA, *P* < 0.001, with controlling for cage effects); the MDD mice were distinguishable from the CD− mice, with the CD+ appearing intermediate to these groupings (Figure 4B; PERMANOVA, *P* < 0.01, with controlling for cage effects). In a comparison of discriminating microbial taxa with FDR < 0.1, we found a higher abundance of Bacteroidales and Clostridiales and a lower relative abundance of taxa within Lactobacillales and Erysipelotrichaceae species in the jejunum of CD− mice compared with all other groups (**Supplemental Figure 2**). No other taxa were significantly different between the CD− and MDD− mice nor were any taxa significantly different between the CD+ and MDD+ mice (FDRs > 0.1).

In stools, we observed a similar trend with greater α-diversity in CD− mice than in other groups [*P *< 0.05, data not shown; CD− (*n* = 5), CD+ (*n* = 5), MDD− (*n* = 4), MDD+ (*n* = 5)]. Also, the CD− mice were differentiable by the weighted and unweighted UniFrac distance metrics on the stool microbiome (PERMANOVA, *P* values <0.01, controlling for cage effects). Many of the same OTUs differentiated these mice as observed for the jejunum as well as an OTU belonging to the genus *Oscillospira* under the order Clostridiales, and several other taxa within the same groups as reported for the jejunum. As before, members of Bacteroidales and Clostridiales were enriched in CD− mouse stool and members of Lactobacillales and Erysipelotrichaceae were depleted (data not shown). No taxa were significantly different between the stools of CD− and MDD− mice nor CD+ and MDD+ mice (FDRs > 0.1, data not shown).

### MDD enteroids exhibit altered crypt domain morphology and intestinal stem cell marker and differentiation gene expression

To determine the extent to which folate and choline deficiency directly affect crypt and intestinal stem cell dynamics ex vivo, we evaluated: *1*) the morphology of crypt domains in murine jejunal enteroids under MDD conditions and *2*) the effects of folate and choline deficiency on crypt morphology (see Figure 5). Jejunal enteroids grown in MDD media were viable but showed morphological differences relative to enteroids grown in standard media after 2 passages, with narrower crypt domains and fewer buds per crypt (*P *< 0.05, Figure 5C, D). Our qualitative impression of crypt domains in deficient media was an increased crypt:villus length ratio, suggesting a relative crypt hypertrophy. Because this hypertrophy might represent a deficit or altered differentiation of intestinal stem cells under folate- and choline-deficient conditions, we measured transcription of genes important for the commitment and differentiation of intestinal epithelial stem cells. As shown in **Table 1**, intestinal stem cell markers, including *Ascl2* (*P* = 0.0002), *Lrig1* (*P* = 0.0047), and *Sox9* (*P* = 0.009), were decreased in the folate- and choline-deficient enteroids compared with controls. We detected no changes in the mRNA levels of the stem cell markers *Lgr5* (*P* = 0.1831), *Bmi1* (*P* = 0.7530), and *Hopx* (*P* = 0.4347). Expression of *Atoh1* (a secretory lineages marker) was upregulated (*P* = 0.003) in folate- and choline-deficient media. In contrast, we observed no change in the levels of the secretory lineage marker, *Hes1* (*P* = 0.6348). Taken together, these results suggest that folate and choline deficiency decreased the relative abundance of intestinal stem cell populations and increased the global secretory progenitor pool, but did not affect the absorptive lineage in enteroids.

**Table 1:** Effects of methyl donor deficient media on mRNA expression of intestinal stem cell genes in enteroids

	Percentage induction		
Gene	Methyl donor regular	Methyl donor deficiency	*P*
*Ascl2*	144.5 ± 14.7	6.5 ± 1.2	0.0002
*Lrig1*	113.3 ± 16.7	73.5 ± 10.1	0.0047
*Bmi1*	9.0 ± 7.1	102.8 ± 35.3	0.7530
*Hopx*	110.5 ± 11.9	97.6 ± 23.7	0.4347
*Lgr5*	99.3 ± 22.9	68.5 ± 22.9	0.1831
*Atoh1*	63.50 ± 9.2	108.8 ± 19.6	0.0305
*Hes1*	72.0 ± 8.5	87.7 ± 25.6	0.6348
*Sox9*	125.8 ± 14.3	52.7 ± 6.7	0.0094
*Klf4*	58.3 ± 4.1	20.0 ± 11.8	0.0181
*Spdef*	46.5 ± 4.2	69.5 ± 17.9	0.2049

Values are means ± SEMs, *n* = 4. Arrows indicate the direction and significance (*P* < 0.05) of the difference. *Ascl2*, Achaete-Scute Family BHLH Transcription Factor 2; *Atoh1*, atonal homolog 1; *Bmi1*, polycomb complex protein BMI-1; *Hes1*, hairy/enhancer of split 1; *Hopx*, HOP homeobox; *Klf4*, Kruppel like factor 4; *Lgr5*, leucine rich repeat containing G protein-coupled receptor 5; *Lrig1*, leucine-rich repeats and immunoglobulin-like domains protein 1; *Sox9*, SRY-box 9; *Spdef*, SAM pointed domain containing Ets transcription factor.

### MDD decreases DNA methylation of genes involved in glycosylation

**FIGURE 6:**
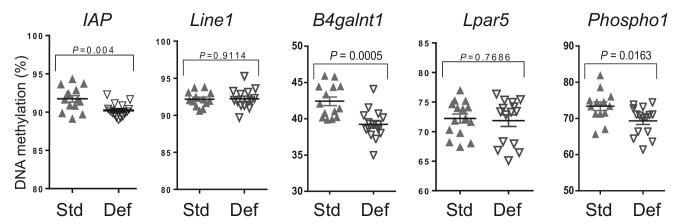
Decreased DNA methylation of glycosylation genes in methyl donor–deficient enteroids. Bisulfite-sequencing DNA methylation levels of generic repetitive elements *IAP* and *Line1* found that the methylation level of *IAP* in MDD and 3’ CGIs of select genes in murine enteroids maintained in either standard or methyl donor–deficient conditions. *B4galnt1*, Beta-1,4-N-Acetyl-Galactosaminyltransferase-1; CGI, CpG-islands; Def, deficient; *IAP*, inhibitor of apoptosis; *Lpar5*, lysophosphatidic acid receptor 5; MDD, methyl donor–deficient diet; *Phospho1*, Phosphoethanolamine/Phosphocholine-Phosphatase; Std, standard.

To explore the hypothesis that combined folate and choline deficiency induces epigenetic effects in the gut epithelium, we compared DNA methylation in enteroids maintained under standard or MDD media conditions for 3 passages. To exclude the possibility that effects on DNA methylation of specific genes were simply due to changes in global DNA methylation, we first analyzed the methylation of 2 generic repetitive elements [inhibitor of apoptosis (*IAP*) and *Line1*] and found that the methylation level of *IAP* in MDD media was decreased relative to control media (*P* = 0.04); however, *Line1* methylation levels were similar in either condition (**Figure 6**). We next analyzed DNA methylation of select genes specifically at 3′ CGIs, a region in which decreased methylation results in decreased gene expression (23). We found that 2 genes related to mucus production, Beta-1,4-N-Acetyl-Galactosaminyltransferase-1 (*B4galnt1*; *P *= 0.0005) and Phosphoethanolamine/Phosphocholine-Phosphatase (*Phospho1*; *P *= 0.020) (Figure 6), exhibited significantly reduced DNA methylation in enteroids maintained in MDD media. We detected no changes in DNA methylation of lysophosphatidic acid receptor 5 (*Lpar5*), a gene involved in sodium and water absorption.

## Discussion

In this series of in vivo and ex vivo MDD (folate and choline) experiments in pregnant mice and their offspring, we observed persistent adverse effects of MDD on the growth and gut health of pups and weanlings. These adverse effects included weight and length faltering, megaloblastic changes of RBCs, modest lengthening of enterocyte nuclei, patchy crypt hypertrophy of the intestinal epithelium, and decreased diversity of SI and fecal microbial communities. We chose to introduce MDD diets to dams during pregnancy based on a previous study by Craciunescu et al. (24), who used a similar experimental design to induce folate deficiency in fetal mice.

Young mice fed MDD diets grew poorly, despite the fact that diets were isocaloric and isonitrogenous. Interestingly, MDD mice without antibiotics displayed hyperphagia, a phenomenon known to occur in situations of extreme calorie restriction (25). This compensatory hyperphagia is thought to increase future risk of obesity and metabolic syndrome in individuals exposed to famine, the well-known “thrifty hypothesis” (26). The absence of hyperphagia in MDD mice administered succinylsulfathiazole raises the possibility that gut microbiota mediate hyperphagia, a hypothesis meriting further study.

Previous animal studies have shown effects of isolated folate deficiency on growth and intestinal morphology. Howard et al. (27) reported that folate-deficient rats (also administered succinylsulfathiazole) were smaller and exhibited irreversible SI crypt hypertrophy when compared with control animals. Similar effects of MDD diets on the microarchitecture of the SI have also been described in more recent studies (28, 29). Our study extends these earlier findings by adding choline deficiency and studying experimental diets both with and without succinylsulfathiazole. Importantly, we observed that succinylsulfathiazole alone was sufficient to induce mild crypt hypertrophy in portions of the SI, even more so than MDD. Succinylsulfathiazole inhibits bacterial folate synthesis, is poorly absorbed, and was used in our experiments to exacerbate dietary MDD. We speculate that folate synthesis by gut microbiota, particularly in the ileum, may be a more readily available and essential source of methyl donor nutrients to intestinal crypts than dietary folate.

Interestingly, MDD resulted in decreased tail length in mice—a surrogate for overall linear growth. Stunting is a marker of chronic malnutrition (30–33). Acute caloric restriction does not typically cause stunting, as usually catch-up growth occurs after normal calorie intake is re-established. The evidence that micronutrient deficiency, in the absence of caloric restriction or macronutrient deficiency, impairs linear growth emphasizes that energy and sufficient micronutrients are necessary in combination for optimal growth.

We found that MDD and antibiotics, alone or in combination, altered the composition of microbial communities in the jejunum and feces of mice. Environmental factors, such as use of antibiotics and dietary components, are well known to influence the composition of the human microbiome (34–36). Several groups have recently shown the influence of different dietary imbalances on intestinal microbiota. Perhaps the most important article on this subject describes the role of the intestinal microbiome and dietary deficiencies in the development of kwashiorkor, one presentation of severe malnutrition (37). Samples of microbiome from Malawian children with kwashiorkor were given orally to adult 8-wk-old mice fed either standard mouse unpurified diet or a low-calorie, nutrient-deficient diet resembling the Malawian diet. The combination of the deficient diet along with the kwashiorkor microbiome resulted in significant weight loss in the mice, and the weight was only partially improved in that group with the reintroduction of a high-calorie diet. These findings suggest that the microbiome is not only affected by the diet, but can interfere with the body's energy harvest capacity, leading to suboptimal utilization of dietary calories and nutrients (38). The same group showed that the result of transplanting microbiota from obese mice to lean germ-free mice is increased fat deposition compared with mice receiving microbiota from lean mice (38). Others have described microbiome shifts (specifically a decrease in Bacteriodetes and an expansion of Proteobacteria and Firmicutes) in mice subjected to a high-fat diet (39).

The evidence for an impact of macronutrients and diet on the gut microbiome is clear; however, the literature on the effects of micronutrients, such as folate and choline, on the intestinal microbiome is more limited. Neither folate deficiency nor supplementation had an effect on the intestinal microbiota in a mouse model of ulcerative colitis (40). In that study, the presence of inflammation was the major factor shaping shifts in the microbiome (40). Spencer et al. (41) aimed to identify microbiome shifts under a choline-deficient environment in 15 healthy adults. Although no discriminatory pattern was identified between subjects, manipulation of choline amounts in the diet led to disturbances in the individual microbiome. Another study described the effect of iron supplementation of infants, suggesting an increase in pathogenic organisms in the group receiving supplemental iron (42). Interactions between vitamin D and the microbiome have been recently described in humans. In a well-designed study by Bashir et al. (43), healthy individuals were given high doses of cholecalciferol (vitamin D_3_) for 8 wk. The composition of intestinal microbiota was examined before and after the intervention by obtaining tissue biopsies from 7 sites throughout the gastrointestinal tract. The results evidenced a significant modulation of the upper gastrointestinal tract microbiome by vitamin D, with a decrease in some pathogenic organisms and an increase in bacterial community richness.

Our in vitro gene expression data suggest that MDD increased the global secretory progenitor pool [atonal homolog 1 (*Atoh1*)] but not the absorptive lineage [hairy/enhancer of split 1 (*Hes1*)]. We further found that SRY box 9 (*Sox9*), a marker related to differentiation and maturation of Paneth cells (44), was decreased with MDD medium. These results are consistent with our in vivo data in which CD+ and MDD+ diets showed a trend towards increased goblet cells and decreased Paneth cells. In addition, our results suggest MDD affects stemness by decreasing slow-cycling stem cell populations such as leucine-rich repeats and immunoglobulin-like domains protein 1 (*Lrig1*) and Achaete-Scute family BHLH transcription factor 2 (*Aslc2*) but not fast-cycling stem cell populations marked by leucine rich repeat containing G protein-coupled receptor 5 (*Lgr5*), polycomb complex protein BMI-1 (*Bmi1*), and HOP homeobox (*Hopx*) (45). Given the importance of folate and choline to DNA synthesis and maturation, these changes likely reflect intestinal stem cell adaption to an environment with reduced methyl donor availability.

An effect of folate-depleted media on global DNA hypomethylation has previously been shown in transformed mouse fibroblasts (NIH3T3) as well as a Chinese hamster ovary line (CHO-K1), but not found in the transformed intestinal epithelial cell lines Caco2 or HCT116 under similar conditions (46). We observed DNA hypomethylation as a result of folate and choline deficiency in enteroids, indicating primary intestinal organoid cultures may be better suited to model the effects of methyl donor nutrients on DNA methylation in intestinal epithelial cells. We found genes involved in the production of mucins (*B4galnt1* and *Phospho1*) were hypomethylated at 3′ CGI, an epigenetic marker related to decrease in gene expression. Of note, *B4galnt1* is normally under transcriptional control by DNA methylation at 3′ CGI and is implicated in inflammatory bowel disease and colorectal cancer (23). Further studies exploring perturbation of the epigenetic program of intestinal stem cells could elucidate mechanisms on how folate and other micronutrients could repair epigenetic pathways leading to EEs.

Our study has several limitations. First, we did not determine whether combined folate and choline deficiency produces more dramatic effects than either deficiency in isolation. Second, similar to other studies, we had difficulty in obtaining accurate choline measurements from plasma to verify deficiency (8, 28). Third, we did not directly assess the effects of MDD diets on the mucus layer, antibacterial peptides, or intestinal barrier function—important aspects of gut health. Lastly, we did not generate enteroids from mice maintained on MDD diets, which might have provided more meaningful data on long-term diet-induced epigenetic changes of intestinal stem cells. As emphasized above, future directions will include efforts to better understand the effects of MDD and antibiotics on gut microbial communities and intestinal stem cell DNA methylation, as well as studies to elucidate mechanistic links between MDD, growth, intestinal epithelial homeostasis, and host–microbe interactions.

In conclusion, dietary MDD impairs early ponderal and linear growth and is accompanied by dysmorphic changes in the crypts of the SI and decreased gut microbial community diversity in mice. In vitro, MDD modulates the expression of gene markers for intestinal stem cells and secretory lineages and decreases the DNA methylation of genes related to mucin production. Further studies are needed to elucidate the mechanisms of these findings and ultimately translate them to methyl donor nutrient–based strategies to promote early gut health.

## Supplementary Material

nzy070_Supplement_Figures_Tables

## Notes

Supported by Bill & Melinda Gates Foundation grant OPP1109785 (to SRM).

Author disclosures: AVAdS, SBdCO, SCDR, LMD, JKR, VSC, HL, EAM, KJB, EA, REL, GAP, LS, and SRM, no conflicts of interest.

Supplemental Tables 1–3 and Supplemental Figures 1 and 2 are available from the “Supplementary data” link in the online posting of the article and from the same link in the online table of contents at https://academic.oup.com/cdn/.

Abbreviations used: BrdU, bromodeoxyuridine; CD, control diet; CD+/−, control diet with/without succinylsulfathiazole; EE, environmental enteropathy; FDR, false discovery rate; MDD, methyl donor deficient/deficiency; MDD+/−, methyl donor–deficient diet with/without succinylsulfathiazole; OTU, operational taxonomic unit; PERMANOVA, permutational multivariate ANOVA; QIIME, Quantitative Insights Into Microbial Ecology; SI, small intestine/intestinal.
