# Supplementary material for: Murine Methyl Donor Deficiency Impairs Early Growth in Association with Dysmorphic Small Intestinal Crypts and Reduced Gut Microbial Community Diversity
Source: Curr Dev Nutr. 2018 Oct 3;3(1):nzy070. doi: 10.1093/cdn/nzy070 (PMC6324351; doi:10.1093/cdn/nzy070)
Supplement: nzy070_Supplement_Figures_Tables [file nzy070_supplement_figures_tables.zip › CDN-D-18-00047_supplementary figure 1.docx]

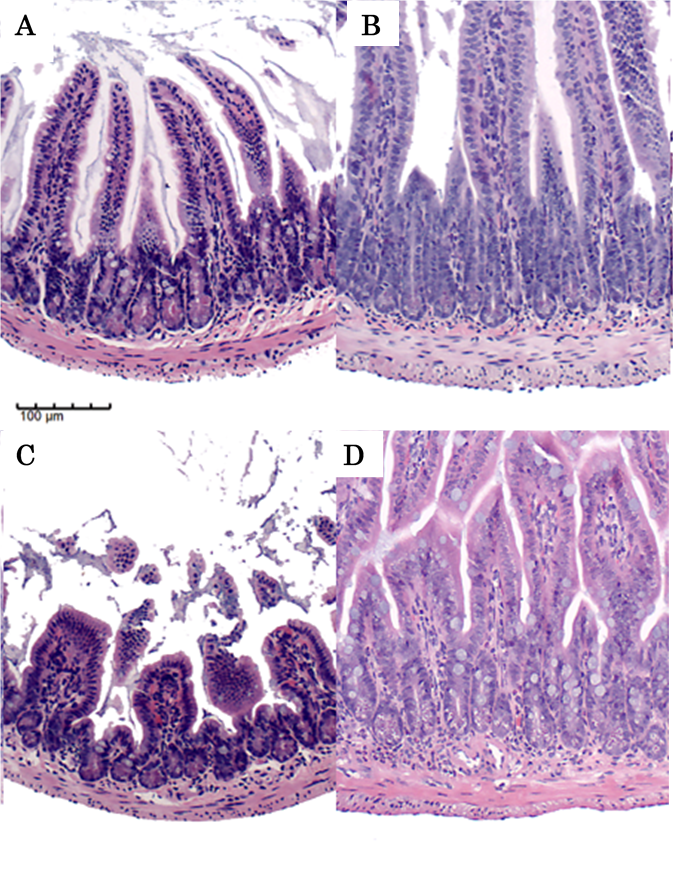


**Supplemental Figure 1. *Methyl donor deficiency and succinylsulfathiazole modulate Paneth and goblet cell abundance in mouse ileum.***  Representative ileal sections from 4 experimental groups: (A) CD-, (B) CD+, (C) MDD-, and (D) MDD+ (1000x magnification). Ileal sections from CD+ (B) and MDD+ (D) mice exhibited a trend towards crypt elongation, increased goblet cells, and decreased Paneth Cells vs. (C) CD- mice.
