# Supplementary material for: Murine Methyl Donor Deficiency Impairs Early Growth in Association with Dysmorphic Small Intestinal Crypts and Reduced Gut Microbial Community Diversity
Source: Curr Dev Nutr. 2018 Oct 3;3(1):nzy070. doi: 10.1093/cdn/nzy070 (PMC6324351; doi:10.1093/cdn/nzy070)
Supplement: nzy070_Supplement_Figures_Tables [file nzy070_supplement_figures_tables.zip › CDN-D-18-00047_supplementary figure 2.docx]

**
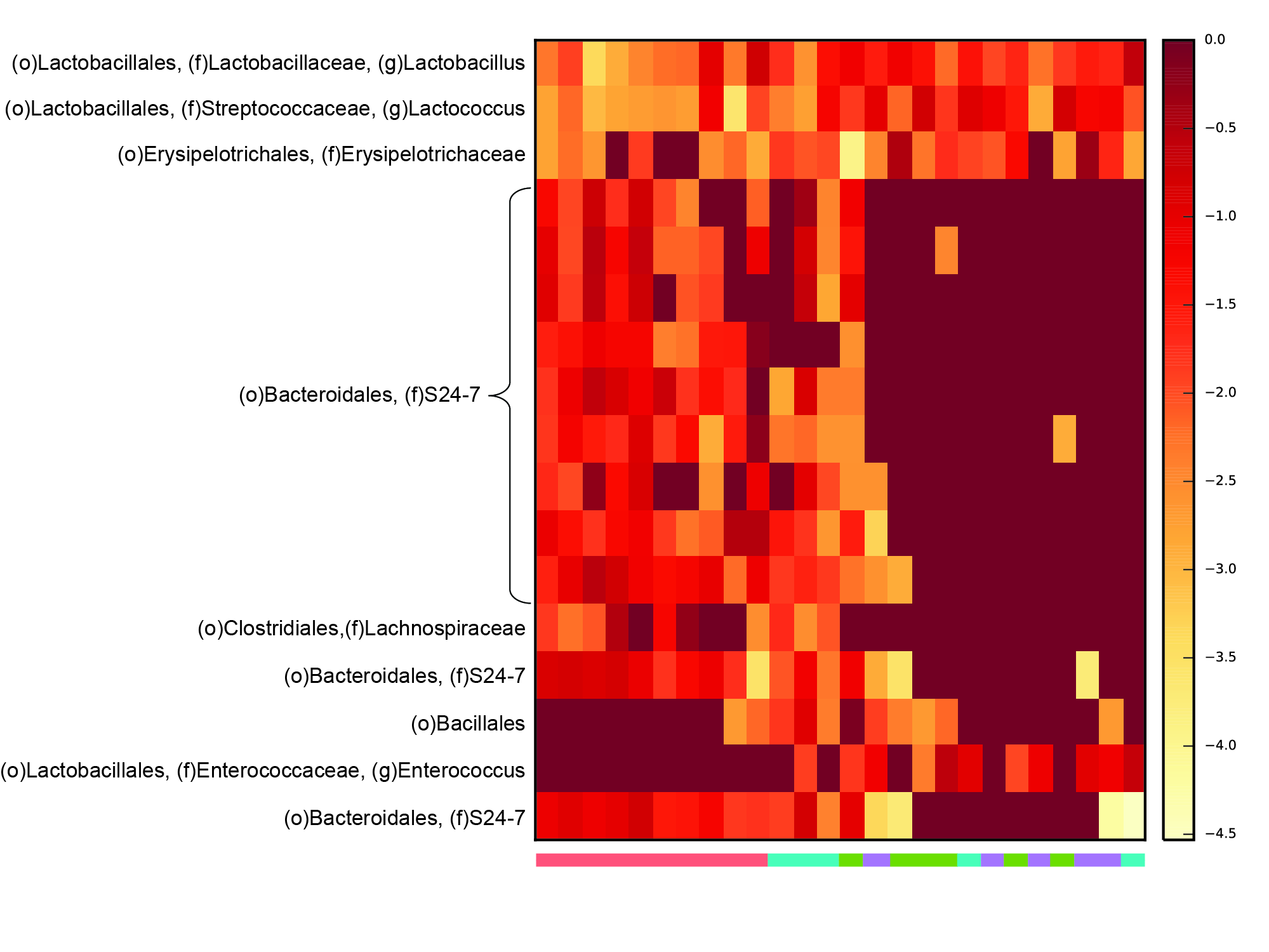
**

**Supplemental Figure 2. *OTUs differentiating CD- mice and other mouse groups*** (FDR<0.10). Taxonomy is indicated: order (o), family (f), genus (g). Mice are shown on the x-axis and colored by diet-treatment group: CD- (red), CD+ (green), MDD- (blue), MDD+ (purple). CD- (N=10); CD+ (N=7); MDD- (N=4); MDD+ (N=5).
