# Supplementary material for: Murine Methyl Donor Deficiency Impairs Early Growth in Association with Dysmorphic Small Intestinal Crypts and Reduced Gut Microbial Community Diversity
Source: Curr Dev Nutr. 2018 Oct 3;3(1):nzy070. doi: 10.1093/cdn/nzy070 (PMC6324351; doi:10.1093/cdn/nzy070)
Supplement: nzy070_Supplement_Figures_Tables [file nzy070_supplement_figures_tables.zip › CDN-D-18-00047_supplementary table 1.docx]

**Supplemental Table 1.** Composition and energy content of experimental diets

| Ingredients | g/Kg | | | |
| --- | --- | --- | --- | --- |
|  | **CD-** | **CD+** | **MDD-** | **MDD+** |
| Casein | 200 | 200 | 200 | 200 |
| L-Cystine | 3 | 3 | 3 | 3 |
| Corn starch | 346 | 346 | 346 | 346 |
| Maltodextrin10 | 45 | 45 | 45 | 45 |
| Dextrose | 250 | 250 | 250 | 250 |
| Cellulose, BW200 | 75 | 75 | 75 | 75 |
| Inulin | 25 | 25 | 25 | 25 |
| Soybean Oil | 70 | 70 | 70 | 70 |
| Potassium citrate 1H2O | 16.5 | 16.5 | 16.5 | 16.5 |
| Calcium carbonate | 5.5 | 5.5 | 5.5 | 5.5 |
| Dicalcium phosphate | 13 | 13 | 13 | 33 |
| Mineral mix S10026 ^a^ | 10 | 10 | 10 | 10 |
| Vitamin Mix V10001 ^b^ | 10 | 10 | 0 | 0 |
| Vitamin Mix V14901 (no folate) c | 0 | 0 | 10 | 10 |
| Choline bitartrate | 2 | 2 | 0 | 0 |
| Succinylsulfathiazole | 0 | 10 | 0 | 10 |
| Nutritional Values |  |  |  |  |
| Protein, % energy | 19 | 18.8 | 19 | 18.8 |
| Carbohydrate, % energy | 63 | 62 | 62.3 | 61.7 |
| Fat, % energy | 6.5 | 6.5 | 6.5 | 6.5 |
| Energy content, (MJ/Kg) | 15.7 | 15.7 | 15.7 | 15.7 |

CD-, control Diet with antibiotic; CD+, control diet with antibiotic; MDD-, methyl Donor deficient diet without antibiotic; MDD+, methyl Donor deficient diet with antibiotic

^a^ Mineral mix (S10026; Research Diet) containing (g/kg): NaCl 259, MgO 41.9, MgSO_4_ 258, (NH_4_)_6_Mo_7_O_24_・4H_2_O 0.3, CrKO_8_S_2_ 1.93, CCuO_3_ 1.05, C_6_H_5_FeO_7_ 21, MnCO_3_ 12.3, KIO_3_ 0.035, NaF 0.2, Na_2_O_3_Se 0.035, and Co_3_Zn 5.6.

^b^ Vitamin mix (V10001; Research Diet) containing (g/kg): retinyl palmitate 0.8, cholecalciferol 1, all-rac-α-tocopheryl acetate 10, menadione sodium bisulfite 0.08, biotin 2, cyanocobalamin 1, folic acid 0.2, nicotinic acid 3, calcium panthotenate 1.6, pyroxidine HCl 0.7, riboflavin 0.6, and thiamin HCl 0.6.

^c^ Vitamin mix (V14901; Research Diet), contain the same composition of Vitamin mix V10001, minus folic acid.
