# Supplementary material for: Murine Methyl Donor Deficiency Impairs Early Growth in Association with Dysmorphic Small Intestinal Crypts and Reduced Gut Microbial Community Diversity
Source: Curr Dev Nutr. 2018 Oct 3;3(1):nzy070. doi: 10.1093/cdn/nzy070 (PMC6324351; doi:10.1093/cdn/nzy070)
Supplement: nzy070_Supplement_Figures_Tables [file nzy070_supplement_figures_tables.zip › CDN-D-18-00047_supplementary table 2.docx]

**Supplemental Table 2.** Commercial primers used for quantitative real-time PCR (Bio-Rad Laboratories)

| Gene | Unigene | Assay identification |
| --- | --- | --- |
| *Spdef* | Mm.286407 | qMmuCED0046601 |
| *Sox9* | Mm.286407 | qMmuCED0044685 |
| *Lrig1* | Mm.245210 | qMmuCID0013023 |
| *Klf4* | Mm.4325 | qMmuCED0045416 |
| *Hprt* | Mm.299381 | qMmuCED0045738 |
| *Hopx* | Mm.181852 | qMmuCID0007483 |
| *Hes1* | Mm.390859 | qMmuCED0045734 |
| *Bmi1* | Mm.289584 | qMmuCED0040846 |
| *Ascl2* | Mm.196417 | qMmuCED0001620 |
| *Actb* | Mm.391967 | qMmuCED0027505 |
