# Supplementary material for: Murine Methyl Donor Deficiency Impairs Early Growth in Association with Dysmorphic Small Intestinal Crypts and Reduced Gut Microbial Community Diversity
Source: Curr Dev Nutr. 2018 Oct 3;3(1):nzy070. doi: 10.1093/cdn/nzy070 (PMC6324351; doi:10.1093/cdn/nzy070)
Supplement: nzy070_Supplement_Figures_Tables [file nzy070_supplement_figures_tables.zip › CDN-D-18-00047_supplementary table 3.docx]

**Supplemental Table 3.** Bisulfite-pyrosequencing primers and sequenced regions for quantitative DNA methylation analysis

| Gene | Foward Primer | Reverse Primer (5' Biotinylated) | Sequence Primer | Sequenced Region |
| --- | --- | --- | --- | --- |
| *Line1* | TGATAGTTTTTGG  AATAGGTAGAAGT | CCCAAATAATACAAACTCTCACTTAA | TTGAGGTAGTATTTTGTGTG | GGTYGGGGAT AGTYGGTT |
| *IAP* | AAGGAAAGGG  GGAGATGTTGG | CRCCTAAAACRTATCACTCCCTAA | ATTTTATGTGTTTTGTTTTT | TTYGTGAYGT  TAATTYGGTY GATGG |
| *B4galnt1* | GGGGAGAGGGAA  AAAGATGTTAGG | ACTCTCCTCCCCTACCAAAT | ATTTTTTTAGGGTTGGTT GGTGTTTTAAGTAATTATTAAAT GGTTGGAGAAGTTTGTG | YGTAGGTYGG AATTTGGYGG T  AYGTGTTGTG GGTGGAYGAY  GATTTTGTTT TTAYGGYGYG TA  GAYGTGTTYG AGAAGAYGTT |
| *Lpar5* | TGGTTTTTTTTA  GGTTTTTTGTGTTAG | ACCTACCCTTCCACAATTC | GTGTGGGTTTTTATTTTGT | TGTTYGTTGT GTTYGTYGTT Y  GYGTGTATA GTTYGTTTTA |
| *Phospho1* | GAAGTAGGAGTTTTG  TTTGGTTATGAA | CTTCCCCCTACTCCCAAAA | TTGGTTATGAATTGTAATAAA GTTGTATGTATTTATTGTAGTAGT | TYGTTTATGT TTGGYGATAG GGGGATGGTT TYGTAGATAG T TTYGYGATA GGTGGTAYGT AGGTTTTYGG GTAGTTGTTG |
